# Training Nurses for Disasters: A Systematic Review on Self-Efficacy and Preparedness

**DOI:** 10.3390/healthcare13243323

**Published:** 2025-12-18

**Authors:** Monica Nikitara, Amarachi Kalu, Evangelos Latzourakis, Costas S. Constantinou, Venetia Sofia Velonaki

**Affiliations:** 1 Department of Health Sciences, School of Life and Health Sciences, University of Nicosia, Nicosia 2417, Cypruslatzourakis.e@unic.ac.cy (E.L.); 2 Department of Basic and Clinical Sciences, Medical School, University of Nicosia, Nicosia 2417, Cyprus; 3 School of Nursing, National and Kapodistrian University of Athens, Goudi, 11 517 Athens, Greece

**Keywords:** disaster self-efficacy, emergency response, simulation-based training, disaster care, nursing students, healthcare education

## Abstract

Background: The rising frequency and complexity of disasters underscores the urgent need for robust preparedness in healthcare. Nurses and nursing students, as key frontline responders, often lack sufficient training to respond effectively to emergencies and recovery efforts. Aim: This review evaluates the effectiveness of disaster preparedness training in terms of nurses’ and nursing students’ self-efficacy in providing disaster care and determines which training approaches are most effective. Method: A systematic review was conducted of peer-reviewed articles published in English between 2014 and 2025 across Medline, PubMed, ProQuest, and Health & Medical Col. Search terms included nurses, nursing students, self-efficacy, disaster training, emergency preparedness, training, simulation and scenario-based learning. Results: Nineteen peer-reviewed studies met the inclusion criteria. Overall, disaster preparedness training was found to enhance nurses’ and nursing students’ self-efficacy, knowledge and skills, with simulation-based and scenario-driven approaches producing the most consistent gains. These methods provided realistic and immersive experiences that fostered confidence and strengthened preparedness. Traditional lectures and workshops also improved outcomes but were generally less effective in sustaining self-efficacy over time. Reported challenges included limited faculty expertise, insufficient institutional support, and psychological barriers that may reduce engagement and impact. Conclusion: Integrating disaster preparedness into nursing curricula and professional training is vital for strengthening nurses’ and nursing students’ self-efficacy in crisis response. Evidence shows that simulation-based education, particularly when combined with traditional approaches, is especially effective in building knowledge and skills. Embedding these methods into training frameworks offers a sustainable strategy to ensure a more competent and resilient nursing workforce.

## 1. Introduction

The frequency and magnitude of natural and man-made disasters are rising globally, posing significant challenges to communities and healthcare systems. Over the past decade, disasters have increased by 60%, leading to substantial loss of life, injury, and displacement [1,2]. These events, ranging from pandemics to natural disasters, have far-reaching social, economic, and environmental impacts, including long-term consequences such as psychological trauma and disrupted medical services [3]. Given the critical role of healthcare in disaster response, the growing trend underscores the necessity of strong disaster preparedness.

A natural disaster is an abrupt, catastrophic event that happens because of Earth’s natural processes, like biological, geological, or meteorological dangers, and causes property damage and human casualties [4]. Man-made disasters are occurrences that cause major disruption and harm to society that are caused by human intent, carelessness, or error, or by a malfunction in a system that was created by humans [5]. Nurses, as the largest segment of the global healthcare workforce, are pivotal in disaster management. Their diverse skill set and frontline status enable them to deliver direct patient care and support community recovery [6]. However, disasters require nurses to possess both general clinical skills and specialized disaster response competencies, including adaptability to resource-limited environments, rapid decision-making, and resilience under stress [7]. Despite their importance, studies reveal that nurses and nursing students are often inadequately trained for disaster scenarios. Low confidence, insufficient training, and a lack of institutional support are common barriers to preparedness [3,8].

A study by Nilsson et al. (2016) highlighted that while registered nurses report higher self-perceived disaster nursing competence than nursing students, workplace exposure to real-world emergency situations significantly influences their preparedness [9]. This underscores the importance of experiential learning in bridging the gap between theoretical knowledge and practical application. Similarly, structured disaster preparedness programs have been shown to significantly improve nursing students’ knowledge and practical skills. However, they noted that while knowledge retention was high, attitudinal shifts were limited, emphasizing the need for continuous reinforcement and hands-on training.

Frameworks such as the WHO and ICN Disaster Nursing Competencies offer comprehensive guidelines to equip nurses with essential skills. Yet, their integration into nursing education remains inconsistent, with fewer than 50% of nursing programs globally incorporating disaster training [6]. A notable gap is the underutilization of simulation-based and experiential learning methods, such as drills, tabletop exercises, and disaster simulations. These methods enhance critical thinking, decision-making, and teamwork, which are crucial in emergencies [3]. However, overcrowded curricula and resource constraints often hinder their inclusion in nursing programs.

Despite these obstacles, progress is being made. Programs integrating competency-based frameworks, interdisciplinary collaboration, and simulation-based learning have shown promise in enhancing disaster response capabilities [6]. The ICN Framework for Disaster Nursing Competencies, for example, provides a structured approach to training nurses in preparedness, response, recovery, and prevention [3]. Additionally, online refresher courses and train-the-trainer programs have demonstrated the potential to expand disaster education across diverse healthcare settings. Al Thobaity et al. (2016) emphasized that standardized competency assessment tools could further strengthen nursing education by identifying key gaps and tailoring training programs to meet regional needs [10].

As the frequency of disasters continues to rise, ensuring that nurses are adequately prepared remains a priority. Strengthening disaster nursing education through evidence-based training programs, policy support, and institutional investment is essential. By addressing educational gaps, overcoming institutional barriers, and leveraging competency-based frameworks, the global nursing workforce can be better equipped to respond to the ever-growing challenges posed by disasters. Therefore, the aim of this study is to evaluate the effectiveness of disaster preparedness training in terms of nurses’ and nursing students’ self-efficacy, knowledge and skills in providing disaster care and to determine which training approaches are most effective. The specific objectives are to assess how structured programs improve knowledge and skills, to compare the impact of simulation-based versus traditional training methods, and to explore factors influencing self-efficacy to provide disaster care.

## 2. Methods

This systematic review was conducted using the PRISMA guidelines to ensure transparency and accuracy in the selection and evaluation of the literature. Prospero Registration Number: CRD420251241507.

### 2.1. Eligibility Criteria

Table 1 below provides a summary of the inclusion and exclusion criteria that were applied to choose pertinent articles.

### 2.2. Information Sources and Search Strategies

The databases ProQuest Central, MEDLINE, Nursing & Allied Health Database, CINAHL, and Scopus were utilized to find pertinent literature. These databases were picked because they contain a wide range of issues related to disaster preparedness, nursing education, and healthcare. Although other databases were taken into consideration, they were not included because of substantial content overlap or lack of relevance to the study’s goals. The University of Nicosia Library federated search platform automatically merges identical records retrieved from multiple databases, so the number of initial records (*n* = 219) already reflects system-level deduplication before manual screening.

The search strategy combined keywords and comparable MeSH terms for “disaster training”,” “nurses,” “effectiveness,” and “simulation”. The search was organized using the Boolean operators OR and AND. Database-specific search strings are shown in Table 2 and Appendix B.

Only peer-reviewed, full-text, English-language journal articles published between 2014 and 2025 were included in the search results. The search was performed on 2025 and covered records published from 1 January 2014 to 30 July 2025. The study did not include studies that addressed disaster-related training or that concentrated on healthcare providers other than nurses.

### 2.3. Study Selection Process

Following the first search over a few chosen databases, all of the records found were imported into a reference management program, where duplicates were eliminated. The eligibility criteria were used to determine the relevancy of the remaining publications’ titles and abstracts.

After that, full-text articles were evaluated to see if they satisfied the requirements for inclusion. Studies that particularly targeted disaster preparedness education for nurses or nursing students were chosen. Articles that were opinion-based, concentrated on non-nursing professionals, or lacked any kind of emergency or catastrophe training were disqualified.

**Figure 1:**
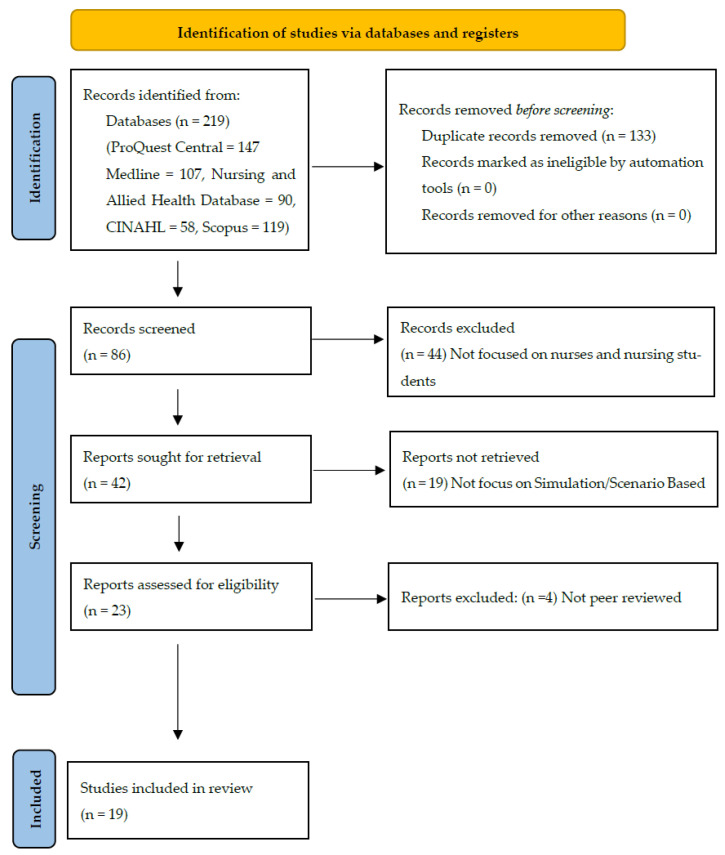
PRISMA flowchart with the search strategy of the systematic review.

To maintain objectivity, two researchers carried out the screening and selection on their own. The PRISMA flow diagram (see Figure 1) shows how the entire selection process adhered to the PRISMA guidelines.

### 2.4. Data Collection Process

A structured form created by the researchers was used to retrieve data from the included studies. Particularly, the study title, authors’ names, year of publication, study purpose, methodology, study design, and an overview of the key conclusions and outcomes were gathered.

Every article was carefully examined, and pertinent information was manually jotted down and arranged in a summary table to aid in theme analysis and comparison. Finding reoccurring trends and assessing the success of disaster training programs for nurses and nursing students were made possible by the information that was retrieved.

Before being applied to the entire collection of included articles, the data extraction procedure was tested on a limited number of studies to guarantee uniformity and reduce error.

### 2.5. Data Items

For each included study, background and context were gathered by gathering data on the study title, authors, year of publication, country of origin, and study design. Additionally, information about the sample size and participant attributes, including training background, nursing role, and academic level, were retrieved. Together with the primary outcomes assessed, including gains in knowledge, skills and self-efficacy, each study’s particular educational approach was also recorded. Comparing various training approaches or control groups was also documented when appropriate.

### 2.6. Studies’ Risk of Bias Assessment

Based on the study design, the Joanna Briggs Institute (JBI) Critical Appraisal Tools were used to evaluate the risk of bias in the included studies. This made it easier to assess the methodological quality and pinpoint any shortcomings.

### 2.7. Effect Measures

The effect measures included changes in knowledge, skills, preparedness, was-efficacy in providing disaster care. Most studies used pre- and post-test assessments with statistical comparisons (e.g., *p*-values, regression coefficients), while some incorporated qualitative feedback. These measures captured the impact of simulation-based and scenario-driven training on disaster preparedness outcomes in nurses and nursing students.

### 2.8. Synthesis Methods

Key patterns and topics pertaining to nursing disaster preparedness were identified, coded, and arranged as part of the thematic synthesis analysis of the chosen studies’ findings by two authors. The findings of each study were examined to extract relevant concepts, which were then grouped into broader thematic categories: training effectiveness and influencing factors, simulation-based learning, and the enhancement of self-efficacy.

### 2.9. Reporting Bias Assessment

The review focused on peer-reviewed, full-text articles published between 2014 and 2025 and included studies from a variety of databases and geographical regions to lower the possibility of reporting bias. To guarantee equal representation, efforts were made to incorporate a variety of training methods and study designs. However, because the review did not include unpublished studies or studies written in languages other than English, publication bias might still exist.

## 3. Results

### 3.1. Study Selection

A total of 219 records were initially identified (ProQuest Central = 147, Medline = 107, Nursing and Allied Health Database = 90, CINAHL = 58, Scopus = 119). After removing duplicates, 86 records remained. Following title and abstract screening, 23 full-text papers were assessed for eligibility, of which 4 were excluded. A total of 19 studies met the inclusion criteria.

The flow diagram (Figure 1) shows how the complete research selection procedure was carried out in compliance with the PRISMA 2020 standards [11].

### 3.2. Study Characteristics

Nineteen research studies (Table A1) published between 2014 and 2025, representing diverse geographical regions including the United States, Iran, Indonesia, South Korea, Turkey, and Spain, were included in the final review. The studies included in this review were predominantly quasi-experimental, with a smaller number of randomized controlled trials and only a few cross-sectional designs. Participants primarily included registered nurses and nursing students, with some studies also involving nursing educators. Sample sizes ranged from fewer than 50 participants to more than 300. Disaster training interventions varied widely in format and delivery, encompassing tabletop exercises, online modules, workshops, simulation-based training, and integrated curriculum models.

### 3.3. Risk of Bias in Studies

The Joanna Briggs Institute (JBI) Qualitative Assessment and Review Instrument Critical Appraisal Checklist was applied to evaluate study quality. This tool assesses methodological rigor and potential bias in design, implementation, and analysis. All studies were appraised using the JBI tools and generally demonstrated adequate methodological quality, though several had notable limitations (Table 3, Table 4 and Table 5).

## 4. Findings

This systematic literature review is structured according to a thematic analysis and is organized into four main areas: the effectiveness of structured disaster training programs, simulation- and scenario-based approaches to disaster training, competency-based disaster nursing education, and factors influencing disaster preparedness.

### 4.1. Effectiveness of Structured Disaster Training Programs

Four studies consistently demonstrated that structured disaster training enhances preparedness, knowledge, and self-efficacy among nursing students and professionals. Koca et al. (2020) evaluated a structured program based on the Jennings Disaster Nursing Management Model with 235 nursing students in Turkey [13]. Significant improvements were observed in preparedness perceptions (+ 33.1%) and self-efficacy (+ 31.7%). Similarly, Xia et al. (2019) tested a seven-hour program in China with 63 students and found significant gains in disaster knowledge and skills, though changes in attitudes were limited [19]. Kalanlar (2018) integrated a disaster nursing module into the curriculum of 150 Turkish students, reporting a sharp rise in confidence (from 23.3% pre-training to 88% post-training) [14]. Finally, Lin et al. (2024) conducted a randomized controlled trial with 100 nurses in Taiwan, showing sustained improvements in readiness up to 12 weeks after a two-day intensive program [12]. Collectively, these findings highlight that structured and scenario-based disaster training substantially improves nursing students’ and professionals’ preparedness, with benefits extending beyond immediate knowledge gains.

### 4.2. Simulation- and Scenario-Based Approaches to Disaster Training

Six studies highlighted the value of experiential learning through simulation and real-life scenarios in disaster preparedness. Hung et al. (2021) investigated a comprehensive 45 h disaster preparedness course with 157 nursing students, combining theoretical instruction with experiential components such as mass casualty simulations, tabletop exercises, simulation-based labs, and disaster management board games [3]. Quantitative analysis revealed significant gains in disaster knowledge and perceived competence, while qualitative findings highlighted improvements in motivation, critical thinking, and self-confidence. Similarly, Ghahremani et al. (2022) compared simulation with traditional workshops in preparing 40 nursing students for bioterrorism response [20]. Although both groups showed improvements, the simulation group achieved superior performance in knowledge retention and practical skills, underscoring the added value of immersive learning methods for high-stakes scenarios.

Park and Hwang (2024) developed a simulation-based disaster nursing program with standardized patients for 140 senior nursing students, demonstrating significant gains in disaster nursing competencies, triage decision-making, preparedness, critical thinking, and confidence [26]. Emaliyawati et al. (2025) tested the Integrated Simulation Enhanced Learning for Disaster Nursing (ISEL-DN) model in a quasi-experimental study with 94 students, showing significantly greater improvements in knowledge (*p* < 0.001) and satisfaction (*p* = 0.026) compared to the control group, although differences in attitudes and self-confidence were not statistically significant [15].

Hsiao et al. (2024) evaluated an immersive cinematic escape room (ICER) with 115 nurses and found significantly greater improvements in disaster preparedness compared to traditional teaching, particularly in the emergency-response domain (β = 9.77, *p* < 0.001; β = 7.83, *p* = 0.013), while both groups showed increases in self-efficacy over time [16]. Phan et al. (2025) integrated an innovative escape room and unfolding preparedness simulation into a community health nursing course with 29 students, significantly increasing knowledge and confidence, with participants reporting high engagement, improved teamwork, and stronger epidemiological understanding, though further refinement was needed to clarify nursing roles during simulations [21]. Hill et al. (2025) implemented a disaster simulation with undergraduate nursing students and found that participation significantly enhanced students’ confidence, preparedness, and perceived ability to respond effectively in real disaster scenarios, reinforcing the role of simulation in building both competence and self-efficacy [17]. The study also shows how disaster simulations not only build technical skills but also reveal the emotional challenges of patient care in crises, reinforcing the importance of resilience-building in training.

These studies provide strong evidence that simulation-based and scenario-driven approaches not only enhance disaster-related knowledge but also foster essential clinical competencies, satisfaction, confidence, decision-making skills, and resilience. Their integration into nursing curricula is therefore recommended to ensure students are better equipped to manage complex and unpredictable disaster situations.

### 4.3. Competency-Based Disaster Nursing Education

Three studies reinforce the importance of competency-based education in strengthening disaster nursing preparedness. Huh S-S, & Kang H-Y (2019) tested a four-week program based on the International Council of Nurses’ disaster nursing competency framework with 60 Korean nursing students [18]. The intervention led to significant gains in knowledge, triage skills, and emergency readiness, confirming the value of structured competency-based training. Park and Kim (2017) surveyed 231 emergency nurses in South Korea to identify factors influencing disaster nursing competencies [24]. Disaster-related experience and knowledge were the strongest predictors, though overall competency levels remained moderate. The findings highlight the need for standardized education, hands-on simulation, and interprofessional training to address persistent gaps. In Saudi Arabia, Al Thobaity et al. (2016) developed and validated a disaster nursing competency scale with 132 emergency nurses [10]. The study identified three key domains—core competencies, barriers to disaster preparedness, and nurses’ roles in disaster management. Despite high reliability scores across domains, significant barriers emerged, including inadequate education, lack of training opportunities, limited expert staff, and insufficient institutional support. Together, these studies underline the necessity of competency-based frameworks to improve nurses’ disaster preparedness. They also call for integrating standardized curricula, expanding training opportunities, and addressing systemic barriers to ensure nurses can respond effectively to large-scale emergencies.

### 4.4. Factors Influencing Disaster Preparedness and Response Willingness

Although structured training improves knowledge, willingness and actual preparedness are influenced by multiple factors. Kang et al. (2022) surveyed 163 nursing students in South Korea and found positive correlations between disaster awareness, willingness to respond, and nursing competency [22]. However, awareness alone did not directly translate into preparedness or competency, underscoring the need for comprehensive disaster education. Nilsson et al. (2016) assessed disaster nursing competence among 569 nursing students and 227 registered nurses in Sweden [9]. Registered nurses, particularly those with emergency care experience, reported significantly higher competence, suggesting that workplace exposure to critical incidents enhances readiness and application of disaster principles. Similarly, Tzeng et al. (2016) evaluated 311 hospital nurses in Taiwan and reported overall poor disaster readiness, particularly in self-protection and emergency response [25]. Prior training, previous disaster experience, and emergency or military backgrounds were key predictors of higher preparedness. It is apparent that while disaster education is essential, preparedness and willingness to respond are strongly shaped by prior experience, clinical background, and targeted training. This highlights the need for both curricular integration and ongoing professional development to ensure effective disaster response across nursing populations. Studies have identified systemic and institutional barriers that hinder effective disaster preparedness among nurses. Azizpour et al. (2022) assessed 472 hospital and pre-hospital emergency nurses in Iran and found generally low levels of disaster preparedness knowledge. Training, age, and prior disaster experience were significant predictors of preparedness, with stronger knowledge correlating positively with better triage decision-making skills. The findings point to gaps in institutional training provision that directly affect clinical decision-making during disasters [23]. Jacobs-Wingo et al. (2016) surveyed 7177 nurses across 20 institutions to evaluate preparedness for CBRNE incidents. The study identified widespread training deficiencies and subsequently developed a train-the-trainer curriculum for nurse educators. A pilot implementation showed marked improvement in test scores (from 54% to 89%), underscoring the value of structured and peer-led training models in addressing institutional gaps [6]. Together, these studies highlight the persistent barriers to disaster preparedness at the organizational and policy levels, including inadequate training opportunities, inconsistent curricula, and limited institutional support. Addressing these challenges requires the integration of structured, scalable education, such as scenario-based modules, LMS-supported programs, and peer-led approaches, into both academic curricula and continuing professional development.

Across the included studies, simulation-based and scenario-driven interventions consistently improved disaster preparedness, self-efficacy, confidence, and decision-making skills. Although the interventions varied in duration, complexity, and format, the overall direction of findings was highly consistent, reinforcing the effectiveness of experiential and competency-based disaster training.

### 4.5. Certainty of Evidence

The certainty of the evidence across the included studies was assessed using the GRADE approach, which evaluates study limitations, consistency of findings, precision of effect estimates, directness, and risk of publication bias. Because the review included heterogeneous study designs, mainly quasi-experimental, randomized controlled trials and cross sectionals, the overall certainty of evidence was generally rated as low to moderate. Most studies demonstrated methodological limitations, small sample sizes, and limited generalizability, which reduced confidence in the findings. However, the consistency of positive effects across diverse interventions supports a moderate level of confidence in the conclusion that disaster training enhances knowledge, skills, and self-efficacy among nursing students and nurses.

## 5. Discussion

This review demonstrates that structured, simulation-based, and competency-driven disaster training consistently strengthens nurses’ knowledge, skills, and self-efficacy. However, willingness to respond and long-term preparedness are shaped by additional factors such as prior experience, institutional support, and systemic barriers.

Structured disaster training programs embedded within curricula or delivered as intensive modules have shown clear benefits. Interventions ranging from multi-week courses [13,14] to shorter intensive workshops [12,19] led to measurable improvements in disaster knowledge, triage skills, and confidence. These findings align with global recommendations by the WHO (2008, 2019) and ICN (2009), which emphasize the integration of emergency preparedness into undergraduate nursing education [4,27,28]. Yet, some studies reported limited changes in attitudes [3,19], suggesting that knowledge gains alone do not guarantee behavioral readiness. This underscores the need for repeated reinforcement and opportunities to practice under realistic conditions.

Simulation- and scenario-based education emerged as one of the most effective approaches. High-fidelity simulations, standardized patients, and OSCE protocols were associated with significant improvements in decision-making, teamwork, and critical thinking [3,20,26]. Prior research also shows that exposure to real or simulated emergencies builds confidence and reduces anxiety [9,29]. Nevertheless, barriers such as high costs, insufficient faculty training, and limited infrastructure constrain access in many contexts [23,27]. To address these inequities, scalable digital innovations—including mobile apps, online modules, and gamified tools—have demonstrated potential to complement traditional simulations [30], particularly in low-resource settings.

Competency-based frameworks provide a structured basis for aligning disaster education with standardized performance outcomes. Evidence from Huh S.-.S & Kang H.-Y. (2019) [18] and Park & Kim (2017) [24] shows that such approaches improve knowledge, triage, and emergency readiness, while Al Thobaity et al. (2016) identified key domains of competencies and barriers in Saudi Arabia [10]. Despite these advances, overall competency levels remain moderate. Limited disaster-related experience, insufficient institutional support, and inconsistent curricula contribute to gaps in readiness. These findings highlight the importance of aligning education with global competency frameworks (WHO, 2021; ICN, 2009) while ensuring that programs are reinforced through hands-on practice and interprofessional collaboration [4,28].

Factors influencing preparedness and willingness to respond extend beyond formal education. Nursing students and professionals with prior disaster exposure, emergency care experience, or military backgrounds consistently report higher preparedness [9,22,25]. Yet, many nurses still feel unprepared, even after training, unless institutional environments support drills, mentorship, and sustained development. Organizational barriers remain significant. Azizpour et al. (2022) identified inadequate training provision as a predictor of poor preparedness in Iran, while Jacobs-Wingo et al. (2016) documented widespread deficiencies in CBRNE training in New York, addressed only after implementing a structured train-the-trainer model [6,23]. These findings underscore the importance of policy-level action to integrate disaster preparedness into national nursing curricula and accreditation frameworks [4,28].

Overall, the evidence suggests that disaster preparedness education is most effective when delivered as part of a multi-faceted strategy: structured curricula, simulation-based learning, competency-driven frameworks, and institutional support. While short-term improvements in knowledge and confidence are consistently reported, questions remain regarding long-term retention, behavioral application, and sustainability. Methodological limitations, such as reliance on self-reported measures, lack of standardized assessment tools, and limited longitudinal follow-up, further constrain the evidence base [12]. Future research should focus on developing standardized, validated tools, expanding studies in low-resource settings, and incorporating mixed-methods designs to capture both measurable outcomes and lived experiences.

The findings from this review highlight the importance of comprehensive, simulation-enhanced, competency-focused training in equipping nurses and nursing students with essential disaster response competencies. Based on the evidence, several recommendations can be made for nursing educators, healthcare institutions, policymakers, and researchers.

For nursing education, disaster preparedness should be a core component of undergraduate and postgraduate curricula rather than an elective. Curricula must combine theoretical foundations with experiential learning such as simulations, role-play, and scenario-based exercises. Simulation-based approaches have been consistently shown to improve decision-making, critical thinking, and real-time response skills. Where high-fidelity resources are limited, virtual platforms or low-cost alternatives can provide valuable experiential learning opportunities. Faculty development is equally important; many educators report feeling underprepared to teach disaster-related content. Structured workshops, certification programs, and train-the-trainer models should therefore be prioritized to build teaching capacity.

For healthcare institutions and practicing nurses, disaster preparedness must be sustained through continuous professional development. Regular refresher courses, interprofessional simulation drills, and emergency exercises should be integrated into staff training. Clear, accessible protocols are needed to ensure role clarity during crises. In addition, preparedness initiatives should address the psychological demands of disaster response by incorporating resilience-building workshops, mental health support, and structured debriefing sessions.

For policymakers and regulators, disaster preparedness education should be standardized and mandated across nursing programs. National councils and accreditation bodies should embed disaster competencies into professional standards, ensuring consistency and eliminating gaps between institutions. Investments in simulation infrastructure, teaching resources, and faculty development are essential, particularly in low-resource contexts. Public–private partnerships and interagency collaboration among health ministries, emergency response teams, and academic institutions can help create cohesive national frameworks for disaster training.

For researchers, more longitudinal studies are needed to evaluate the long-term impact of disaster training on behavior and real-world performance. Greater attention should also be given to underrepresented groups, including practicing nurses, rural health workers, and those in high-risk regions. Mixed-methods approaches combining quantitative outcomes with qualitative insights would provide a richer understanding of the effectiveness and challenges of disaster preparedness programs. Future studies could also benefit from established theoretical frameworks. The ICN Disaster Nursing Competency Framework offers a globally recognized model for structuring competencies across prevention, response, recovery, and mitigation. In parallel, Bandura’s Social Cognitive Theory provides a useful lens for exploring behavioral dimensions of disaster readiness, particularly in relation to self-efficacy, motivation, and learning through observation [31].

### Limitations

Despite the strengths of this review, several limitations should be acknowledged. A potential publication bias cannot be excluded, as studies reporting nonsignificant or negative outcomes may be underrepresented in the peer-reviewed literature. Additionally, the English-only inclusion criterion may have led to the omission of relevant studies published in other languages, potentially limiting the geographical scope of the evidence. Methodological limitations within the included studies, such as reliance on self-reported measures, small sample sizes, and varied research designs, also reduce the overall certainty and generalizability of the findings. Future systematic reviews could mitigate these limitations by incorporating non-English databases, assessing gray literature, and exploring meta-analytic approaches where data homogeneity permits, thereby strengthening the comprehensiveness and robustness of the evidence base.

## 6. Conclusions

The increasing frequency and complexity of disasters underscore the urgent need for a nursing workforce that is knowledgeable, confident, and fully prepared to respond. Across the studies reviewed, simulation-based and competency-driven disaster education emerged as the most consistently effective approach, yielding marked improvements in knowledge, triage skills, preparedness, critical thinking, and self-efficacy among both nursing students and practicing nurses (Table A1). High-fidelity simulations, immersive scenarios, escape rooms, standardized patients, and structured multi-module programs all demonstrated significant gains, often outperforming traditional or lecture-based methods and revealing important gaps in role clarity, emotional readiness, and decision-making under pressure. While these findings highlight the clear value of simulation-enhanced training, persistent challenges—including limited institutional resources, inconsistent disaster curricula, and varying levels of psychological preparedness—continue to hinder widespread implementation and sustainability. Scalable hybrid models, technology-supported platforms, and train-the-trainer approaches represent promising strategies for broadening access, particularly in resource-limited settings. To strengthen the field, future work should prioritize standardized outcome measures, longitudinal follow-up, and stronger policy and organizational support for disaster education. Ultimately, embedding structured, simulation-rich, competency-based disaster preparedness training across all levels of nursing education and professional practice is essential to equip nurses to respond effectively, confidently, and compassionately in times of crisis.

## Figures and Tables

**Table 1:** Inclusion and Exclusion Criteria.

Criteria	Inclusion	Exclusion
Publication Years	Published between 2014–2025	Published before 1 January 2014 and after 30 July 2025
Language	Articles published in English	Non-English language articles without translation
Type	Peer-reviewed Research Articles	Not peer-reviewed
Topic Focus	Articles focused on nursing disaster preparedness, education	Studies unrelated to nursing or disaster preparedness (e.g., friendship, military, non-healthcare contexts)

**Table 2:** Search Keywords and Strategies.

Population (P)		Intervention (I)		Comparison (C)		Outcomes (O)
(nursing students OR “student nurses” OR “undergraduate student nurses” OR nurse OR nurses OR nursing OR “nursing staff”)	AND	(“disaster preparedness” OR “disaster response” OR “disaster management” OR preparation OR preparedness OR readiness)	AND	(“simulation training” OR “simulation education” OR “simulation learning” OR “scenario based learning”)	AND	(self-efficacy OR “self efficacy” OR confidence OR “self esteem”)

**Table 3:** JBI Critical Appraisal of Randomized Controlled Trials.

Author (Year)	Q1	Q2	Q3	Q4	Q5	Q6	Q7	Q8	Q9	Q10	Q11	Q12	Q13
Lin et al. (2024) [12]	√	√	√	√	No	X	No	√	√	√	√	√	√
Koca et al. (2020) [13]	√	√	√	√	No	√	No	√	√	√	√	√	√

**Table 4:** JBI Critical Appraisal of Quasi-Experimental Studies.

Author (Year)	Q1	Q2	Q3	Q4	Q5	Q6	Q7	Q8	Q9
Kalanlar (2018) [14]	√	√	√	No	√	√	√	√	√
Hung et al. (2021) [3]	√	√	√	No	√	√	√	√	√
Emaliyawati et al. (2025) [15]	√	√	√	No	√	√	√	√	√
Hsiao et al. (2024) [16]	√	√	√	No	√	√	√	√	√
Hill et al. (2025) [17]	√	√	√	No	√	√	√	√	√
Huh S-S, Kang H-Yet al. (2019) [18]	√	√	√	No	√	√	√	√	√
Xia et al. (2020) [19]	√	√	√	No	√	√	√	√	√
Ghahremani et al. (2022) [20]	√	√	√	No	√	√	√	√	√
Phan et al. (2025) [21]	√	√	√	No	√	√	√	√	√
Ja-cobs-Wingo et al. (2016) [6]	√	√	√	No	√	√	√	√	√
Al Thobaity et al. (2016) [10]	√	√	√	No	√	√	√	√	√

**Table 5:** JBI Critical Appraisal of Cross-Sectional Studies.

Author (Year)	Q1	Q2	Q3	Q4	Q5	Q6	Q7	Q8
Kang et al. (2022) [22]	√	√	√	√	√	√	√	√
Nilsson et al. (2016) [9]	√	√	√	√	√	√	√	√
Azizpour et al. (2022) [23]	√	√	√	√	√	√	√	√
Park & Kim (2017) [24]	√	√	√	√	√	√	X	√
Tzeng et al. (2016) [25]	√	√	√	√	√	√	√	√
Park & Hwang (2024) [26]	√	√	√	√	√	√	√	√

## Data Availability

All data underlying this systematic review are drawn from previously published sources cited within the manuscript. Processed data are presented in Table A1 and the reference list. Further details are available from the corresponding author upon reasonable request.

## Appendix A

**Table A1:** Studies’ Characteristics.

Authors/Date	Aim	Methodology	Sample	Findings
Emaliyawati, et al. (2025) [15]	To explore the effectiveness of the ISEL-DN model in enhancing knowledge, attitudes, satisfaction, and self-confidence among undergraduate nursing students.	A quasi-experimental study with a control group	94 undergraduate nursing students (Intervention group: 47 and control group: 47)	The intervention group demonstrated significant improvements in knowledge, attitudes, satisfaction, and self-confidence from pretest to posttest (all *p* < 0.05), while the control group showed smaller gains, particularly limited in post-disaster knowledge and attitudes.
Phan, Q. T. et al. (2025) [21]	To implement and evaluate an innovative escape room and unfolding disaster preparedness simulation in a pre-licensure nursing program	Descriptive pilot evaluation design	29 pre-licensure nursing students	The escape room and unfolding disaster simulation improved nursing students’ knowledge (100%), confidence (93–100%), and teamwork (96.6%), though fewer students reported clarity in their roles during the disaster scenario (45–55%).
Hsiao et al. (2024) [16]	To develop and evaluate the effectiveness of an immersive cinematic escape room (ICER) instructional approach in disaster preparedness and self-efficacy in nurses.	Quasi-experimental research design	115 Nurses	The experimental group, lacking prior disaster preparedness education experiences, demonstrated a statistically significant improvement (*p* < 0.01) compared to the control group with more such experiences. At week four, both groups showed improvement in the self-efficacy scores, but the improvement did not achieve statistical significance (*p* > 0.05).
Hill, P. P. et al. (2025) [17]	To evaluate the effects of participating in a large-scale community disaster simulation on nursing students’ disaster preparedness and management competency,	Quasi experimental exploratory study,	senior nursing students (*n* = 44)	Students’ perceived competency dropped significantly after the simulation highlights educational gaps and the need for structured, ongoing disaster curricula. It shows how disaster simulations not only build technical skills but also reveal the emotional challenges of patient care in crises.
Hugh et al. (2019) [18]	To evaluate the impact of a disaster nursing educational program on disaster nursing competencies among Korean nursing students.	A quasi-experimental design was employed, with the experimental group receiving disaster nursing training on prevention, preparedness, response, and recovery stages, measuring knowledge, triage skills, and readiness.	−60 junior nursing students from two nursing colleges in Korea	Results showed significant improvements in the experimental group: knowledge (t = 14.37, *p* < 0.001), triage skills (t = 7.90, *p* = 0.002), and readiness (t = 10.82, *p* < 0.001). The program was effective in enhancing disaster nursing competency and is recommended for nursing education.
Lin, C. et al., (2024) [12]	To assess how well a structured DMTP (disaster management training program) affected nurses’ preparedness for disaster response.	The study divided into experimental and control groups evaluated disaster readiness across emergency response, clinical management, self-protection, and personal preparation at baseline and 12 weeks post-intervention.	100 nurses in Taiwan	The study suggests structured DMTP with diverse teaching methods as an essential part of ongoing nursing education to enhance disaster preparedness across all four domains.
Hung et al. (2021) [3]	To evaluate the effectiveness of a disaster management training course in improving Hong Kong nursing students’ disaster knowledge, willingness, and perceived ability to respond to public health emergencies or disasters.	The study employed a mixed-method design, involving pre- and post-intervention comparisons and qualitative focus group interviews, to evaluate the effectiveness of a 45 h disaster management training course.	A total of 157 nursing students participated and completed the pre- and post-intervention questionnaires.	Concerns were divided into three categories: organizational support, personal risk perceptions, and catastrophe contextual variables. However, there were notable advancements in disaster knowledge and perceived capacity.
Koca, B. et al. (2020) [13]	To examine the impact of a six-module training program, utilizing the Jennings Disaster Nursing Management Model and a learning management system, on nursing students’ disaster preparedness perceptions.	A randomized controlled trial using a two-group comparison design, including an experimental group (EG) and a control group (CG).	Third-year nursing students from a city in western Turkey, with 127 in the experimental group and 108 in the control group.	The training program significantly improved disaster preparedness perceptions and response self-efficacy in the experimental group, while moderately affecting their knowledge and self-efficacy.
Jacobs-Wingo et al., (2016) [6]	To improve emergency preparedness among nurses for chemical, biological, radiological, nuclear, and explosive (CBRNE) events by addressing training and confidence gaps.	The CBRNE curriculum was developed through surveys, focus groups, and training sessions, identifying gaps in emergency preparedness among nursing staff in 20 NYC hospitals.	A study involving 7177 NYC nursing staff, 22 nurse educators, and 11 nurses conducted surveys, focus groups, and pilot training sessions.	The CBRNE curriculum, including six modules, just-in-time training, and an online refresher course, significantly enhanced nurses’ knowledge of CBRNE events, from 54% to 89% post-training.
Bilge Kalanlar (2018) [14]	To assessed the influence of disaster nursing education on undergraduate nursing students’ disaster preparedness and suggested enhancements in disaster preparedness education.	A quasi-experimental study compared knowledge and preparedness of final-year nursing students in a disaster nursing and management module with a control group, achieving a 90% success rate.	The study involved final-year undergraduate nursing students who chose the disaster nursing and management module as their treatment group.	The treatment group demonstrated a significant enhancement in disaster knowledge, preparedness, and management, indicating that the disaster nursing module effectively prepared students for disaster response and recovery.
Park and Kim (2017) [24]	To identify factors influencing the disaster nursing core competencies of emergency nurses.	A survey-based study was conducted using a questionnaire to collect data on disaster-related experience, attitude, knowledge, and disaster nursing core competencies.	The study included 231 emergency nurses working in 12 hospitals in South Korea.	Multiple regression analysis showed that disaster-related experience had the strongest influence on disaster nursing core competencies, followed by disaster-related knowledge. These factors explained 25.6% of the variance in disaster nursing core competencies, with statistical significance (F = 12.189, *p* < 0.001). The study highlighted the importance of education and training programs to improve nurses’ disaster preparedness.
Xia et al., (2020) [19]	To develop and evaluate a disaster nursing preparedness training program to improve nursing students’ knowledge, skills, and family preparedness in disaster situations.	An experimental pretest–posttest control group design was used. Participants were randomly assigned to either the experimental or control group.	63 nursing students (31 in the experimental group, 32 in the control group).	Students in the training program showed greater improvements in knowledge and skills than those in the control group. These improvements remained one month after the intervention. However, there were no significant changes in attitude over time.
Ghahremani et al., (2022) [20]	To compare the effectiveness of simulation and workshop methods in improving nursing students’ knowledge and practice regarding bioterrorism.	An experimental study with pretest and posttest design. Data were collected using a demographic questionnaire, bioterrorism knowledge scale, and OSCE checklist.	40 final-year nursing students, randomly assigned to two groups (20 in simulation, 20 in workshop).	Both groups showed significant improvements in knowledge and performance (*p* < 0.001). However, the simulation group outperformed the workshop group in knowledge and most performance domains.
Nilsson et al., (2016) [9]	To compare self-reported disaster nursing competence (DNC) between nursing students (NSs) and registered nurses (RNs) and explore associations between DNC and background factors.	A cross-sectional study using the 88-item Nurse Professional Competence Scale, including three DNC-related items.	569 nursing students (NSs) and 227 registered nurses (RNs).	Registered nurses (RNs) had higher disaster nursing competence (DNC) than nursing students (NSs), especially in handling violence and applying disaster medicine. RNs in emergency care scored higher than those in other fields. Working night shifts and emergency care experience were linked to better DNC. Exposure to serious events improved nurses’ ability to manage disasters and follow safety rules.
Azizpour, Mehri and Soola, (2022) [23]	To assess disaster preparedness knowledge among hospital and pre-hospital emergency nurses and identify its predictors	A descriptive cross-sectional study conducted on 472 emergency nurses in Ardabil, Iran, using self-reported questionnaires (EPIQ and TDMI). Data were analyzed with SPSS.	472 hospital and pre-hospital emergency nurses, selected through convenience sampling.	Emergency nurses had low disaster preparedness knowledge. Key predictors included triage decision-making, age, residence, prior training, disaster experience, and training organization (*p* < 0.05). Higher knowledge correlated with better triage skills. Improved training is recommended.
Al Thobaity, Williams and Plummer, (2016) [10]	To develop a valid and reliable scale to assess disaster nursing core competencies, roles, and barriers in Saudi Arabia.	A principal component analysis (PCA) was conducted using a self-report questionnaire with 93 items rated on a Likert scale. Data were collected from emergency nurses in two hospitals.	132 emergency nurses (66% response rate) from two hospitals in Saudi Arabia.	PCA identified three key factors after removing 49 redundant items, leaving 44 items that explained 77.3% of the variance. The scale showed high reliability, with Cronbach’s alpha of 0.96 overall, and 0.98, 0.92, and 0.86 for the three factors.
Kang, Lee and Seo, (2022) [22]	To examine nursing students’ disaster awareness, preparedness, willingness to respond, and disaster nursing competency, and their relationships.	A descriptive study with 163 nursing students. Data were analyzed using descriptive statistics and Pearson’s correlation coefficients.	163 nursing students.	Disaster awareness correlated positively with willingness to respond. Disaster preparedness and willingness to respond correlated positively with nursing competency. However, disaster awareness was not significantly linked to preparedness or competency, and preparedness did not correlate with willingness to respond.
Yeon Mi Park and Won Ju Hwang, (2024) [26]	This study aimed to develop and evaluate a simulation-based disaster nursing education program using standardized patients, based on the International Council of Nurses’ Framework of Disaster Nursing Competencies.	A quasi-experimental design with a pretest–posttest control group was used. The program included a 60 min lecture and two simulation scenarios. The effectiveness was measured through disaster nursing competencies, triage skills, preparedness, critical thinking, and confidence.	The study involved 140 senior nursing students from two universities	students in the simulation-based training group showed significant improvements in disaster nursing competencies, triage skills, preparedness, critical thinking, and confidence compared to the other groups (*p* < 0.001). This confirms the effectiveness of simulation-based training in disaster nursing education.
Tzeng et al., (2016) [25]	This study aimed to assess hospital nurses’ perceived readiness for disaster response and the factors influencing their willingness to work outside the hospital during disasters.	A cross-sectional research design was used. Data were collected through a 40-item self-administered questionnaire covering personal preparation, self-protection, emergency response, and clinical management. Statistical analysis included descriptive statistics, independent *t*-tests, and generalized linear models.	The study involved 311 registered nurses from a military hospital in Taiwan.	Most hospital nurses had poor readiness for disaster response. Their preparedness was strongly linked to disaster-related training, prior disaster response experience, and experience in emergency or intensive care settings.

## Appendix B. Search Strings for Databases

**CINAHL:** (nursing students OR “student nurses” OR “undergraduate student nurses” OR nurse OR nurses OR nursing OR “nursing staff”) AND (“disaster preparedness” OR “disaster response” OR “disaster management” OR preparation OR preparedness OR readiness) AND (“simulation training” OR “simulation education” OR “simulation learning” OR “scenario based learning”) AND (self-efficacy OR “self efficacy” OR confidence OR “self esteem”)

**ProQuest Central, Medline, Nursing and Allied Health Database** (nursing students OR “student nurses” OR “undergraduate student nurses” OR nurse OR nurses OR nursing OR “nurs-ing staff”) AND (“disaster preparedness” OR “disaster response” OR “disaster management” OR preparation OR preparedness OR read-iness) AND (“simulation training” OR “simulation education” OR “simulation learning” OR “scenario based learning”) AND (self-efficacy OR “self efficacy” OR confidence OR “self esteem”)

**Scopus:** ((nursing students OR “student nurses” OR “undergraduate student nurses” OR nurse OR nurses OR nursing OR “nursing staff”) AND (“disaster preparedness” OR “disaster response” OR “disaster management” OR preparation OR preparedness OR readiness) AND (“simulation training” OR “simulation education” OR “simulation learning” OR “scenario based learning”) AND (self-efficacy OR “self efficacy” OR confidence OR “self esteem”))

## References

[1] Lim J., Skidmore M. (2025). Natural Disasters: Impacts and Recovery. Oxford Research Encyclopedia of Economics and Finance.

[2] Topluoglu S., Taylan-Ozkan A., Alp E. (2023). Impact of wars and natural disasters on emerging and re-emerging infectious diseases. Front. Public Health.

[3] Hung M.S.Y., Lam S.K.K., Chow M.C.M., Ng W.W.M., Pau O.K. (2021). The Effectiveness of Disaster Education for Undergraduate Nursing Students’ Knowledge, Willingness, and Perceived Ability: An Evaluation Study. Int. J. Environ. Res. Public Health.

[4] World Health Organization (2021). Health Emergency and Disaster Risk Management Framework.

[5] International Federation of Red Cross and Red Crescent Societies (IFRC) (2020). Types of Disasters: Definition of Hazard. https://alnap.org/help-library/resources/ifrc-types-of-disasters/.

[6] Jacobs-Wingo J.L., Schlegelmilch J., Berliner M., Airall-Simon G., Lang W. (2018). Emergency Preparedness Training for Hospital Nursing Staff, New York City, 2012–2016. J. Nurs. Scholarsh..

[7] Ličen S., Prosen M. (2025). Disaster Nursing Competencies in a Time of Global Conflicts and Climate Crises: A Cross-Sectional Survey Study. Int Nurs Rev..

[8] Achora S., Kamanyire J.K. (2017). Disaster preparedness: Need for inclusion in undergraduate nursing education. Nurse Educ. Today.

[9] Nilsson J., Johansson E., Carlsson M., Florin J., Leksell J., Lepp M., Lindholm C., Nordström G., Theander K., Wilde-Larsson B. (2016). Disaster nursing: Self-reported competence of nursing students and registered nurses, with focus on their readiness to manage violence, serious events and disasters. Nurse Educ. Pract..

[10] Al Thobaity A., Williams B., Plummer V. (2016). A new scale for disaster nursing core competencies: Development and psychometric testing. Australas. Emerg. Nurs. J..

[11] Page M.J., McKenzie J.E., Bossuyt P.M., Boutron I., Hoffmann T.C., Mulrow C.D., Shamseer L., Tetzlaff J.M., Akl E.A., Brennan S.E. (2021). The PRISMA 2020 statement: An updated guideline for reporting systematic reviews. BMJ.

[12] Lin C.-H., Tzeng W.-C., Chiang L.-C., Lu M.-C., Lee M.-S., Chiang S.-L. (2024). Effectiveness of a Structured Disaster Management Training Program on Nurses’ Disaster Readiness for Response to Emergencies and Disasters: A Randomized Controlled Trial. J. Nurs. Manag..

[13] Koca B., Arkan G. (2020). The effect of the disaster management training program among nursing students. Public Health Nurs..

[14] Kalanlar B. (2018). Effects of disaster nursing education on nursing students’ knowledge and preparedness for disasters. Int. J. Disaster Risk Reduct..

[15] Emaliyawati E., Ibrahim K., Trisyani Y., Songwathana P. (2025). The Effect of Integrated Simulation Experiential Learning Disaster Nursing for Enhancing Learning Outcomes Among Undergraduate Nursing Students: A Quasi-Experimental Study. Adv. Med. Educ. Pract..

[16] Hsiao C.-C., Huang C.-Y., Lai F.-C., Chen T.-L., Cheng S.-F. (2024). Development and Evaluation of an Immersive Cinematic Escape Room for Disaster Preparedness and Self-Efficacy Among Nurses. Clin. Simul. Nurs..

[17] Hill P.P., Díaz D.A., D’Amato-Kubiet L.A. (2025). Disaster! The Effects of a Large-Scale Simulation on Nursing Students’ Disaster Competence. J. Nurs. Educ..

[18] Huh S.-S., Kang H.-Y. (2019). Effects of an educational program on disaster nursing competency. Public Health Nurs..

[19] Xia R., Li S., Chen B., Jin Q., Zhang Z. (2020). Evaluating the effectiveness of a disaster preparedness nursing education program in Chengdu, China. Public Health Nurs..

[20] Ghahremani M., Rooddehghan Z., Varaei S., Haghani S. (2022). Knowledge and practice of nursing students regarding bioterrorism and emergency preparedness: Comparison of the effects of simulations and workshop. BMC Nurs..

[21] Phan Q.T., Chance-Revels R., Clark-Youngblood M., Baker H., Kimble L.P. (2025). Integrating Disease Investigation Escape Room and Preparedness Simulation into Nursing Education. Disaster Med. Public Health Prep..

[22] Kang J.-S., Lee H., Seo J.M. (2022). Relationship Between Nursing Students’ Awareness of Disaster, Preparedness for Disaster, Willingness to Participate in Disaster Response, and Disaster Nursing Competency. Disaster Med. Public Health Prep..

[23] Azizpour I., Mehri S., Soola A.H. (2022). Disaster preparedness knowledge and its relationship with triage decision-making among hospital and pre-hospital emergency nurses—Ardabil, Iran. BMC Health Serv. Res..

[24] Park H.-Y., Kim J.-S. (2017). Factors influencing disaster nursing core competencies of emergency nurses. Appl. Nurs. Res..

[25] Tzeng W.-C., Feng H.-P., Cheng W.-T., Lin C.-H., Chiang L.-C., Pai L., Lee C.-L. (2016). Readiness of hospital nurses for disaster responses in Taiwan: A cross-sectional study. Nurse Educ. Today.

[26] Park Y.M., Hwang W.J. (2024). Development and Effect of a Simulation-Based Disaster Nursing Education Program for Nursing Students Using Standardized Patients. J. Nurs. Res..

[27] World Health Organization (2008). Integrating Emergency Preparedness and Response into Undergraduate Nursing Curricula: Report on a WHO Meeting.

[28] International Council of Nurses (ICN) (2009). ICN Framework of Disaster Nursing Competencies.

[29] Jose M.M., Dufrene C. (2014). Educational competencies and technologies for disaster preparedness in undergraduate nursing education: An integrative review. Nurse Educ. Today.

[30] Genç F.Z., Yiğitbaş Ç., Uzun Ö. (2025). Effect of structured digital-based education on disaster literacy and preparedness beliefs among nursing students: A randomized controlled study. Nurse Educ. Today.

[31] Kılınç G., Yıldız E., Harmanci P. (2018). Bandura’s Social Learning and Role Model Theory in Nursing Education. https://www.researchgate.net/publication/329814373_Bandura.

